# Emerging Roles of lncRNAs Regulating RNA-Mediated Type-I Interferon Signaling Pathway

**DOI:** 10.3389/fimmu.2022.811122

**Published:** 2022-02-25

**Authors:** Xiaoxin Ji, Wei Meng, Zichuan Liu, Xin Mu

**Affiliations:** ^1^ School of Pharmaceutical Science and Technology, Tianjin University, Tianjin, China; ^2^ Tianjin University and Health-Biotech United Group Joint Laboratory of Innovative Drug Development and Translational Medicine, Tianjin University, Tianjin, China

**Keywords:** lncRNA, type-I interferon, PRR, PAMP, ISG

## Abstract

The type-I interferon (IFN-I) signaling pathway plays pivot roles in defending against pathogen invasion. Exogenous ssRNA and dsRNA could be immunogenic. RNA-mediated IFN signaling is extensively studied in the field. The incorrect functioning of this pathway leads to either autoimmune diseases or suffering from microorganism invasion. From the discrimination of “self” and “non-self” molecules by receptors to the fine-tune modulations in downstream cascades, all steps are under the surveillance featured by complex feedbacks and regulators. Studies in recent years highlighted the emerging roles of long noncoding RNAs (lncRNAs) as a reservoir for signaling regulation. LncRNAs bind to targets through the structure and sequence, and thus the mechanisms of action can be complex and specific. Here, we summarized lncRNAs modulating the RNA-activated IFN-I signaling pathway according to the event order during the signaling. We hope this review help understand how lncRNAs are participating in the regulation of IFN-I signaling.

## Introduction

Cells have intrinsic pathways to fight against pathogen invasion, and interferon (IFN) signaling is one of the most important (1). IFNs are classified into three types based on their amino acid sequences and functions. The type-I IFNs (IFN-I) include IFN-α, -β, -ε, −κ, and -ω, while IFN-γ is the only type II IFN (IFN-II). Type III IFNs are divided into IFN-λ1, -λ2, -λ3 and -λ4 (2). Of these, IFN-I and IFN-III are activated by “non-self” stimuli such as pathogens and directly counteract invasions. IFN-II functions to modulate the immune system. The activation of IFN-I signaling results in other consequences such as cell proliferation arrest, global translational repression, and even cell death (3).

Exogenous ssRNA and dsRNA can be immunogenic and activate IFN-I signaling (4). It could be derived from virus infection, bacterial invasion, or sometimes, *in vitro* transcribed RNAs (4). Host-encoded sensors for non-self RNA include the cytosolic retinoic acid-inducible gene I (RIG-I) and melanoma differentiation-associated protein 5 (MDA5), and the endosomal toll-like receptor 3 (TLR3), TLR7, and TLR8 (will be discussed in more detail below). RIG-I recognizes double-stranded RNAs (dsRNAs) with triphosphates (ppp) or diphosphates (pp) and 2′-O-unmethylated at the 5′-end. Such request excludes endogenous mRNAs, rRNAs, and tRNAs as its ligand (5). MDA5 prefers long dsRNA with a perfect duplex structure. Endogenous duplex structures are always imperfect duplexes and further edited by the host-encoded adenosine deaminase, RNA-specific 1 (ADAR1), which is a dsRNA binding protein and performs A-to-I editing on duplex (6). Such an event melts the integrity of endogenous duplexes and removes them from MDA5 recognition. TLRs face the interior of the endosome, thus cytosolic RNAs are normally kept away from these receptors. Studies from type-I interferonopathies indicated that even the endogenous RNA molecules can be immunogenic when disrupted RNA metabolisms happen because of gene mutations (7). For example, the loss-of-function (LOF) mutation (P193A or G1007R) or deficiency of *ADAR1* causes aberrant IFN-I signaling due to the lack of edition on endogenous inverted-repeat Alu duplexes which then are recognized by MDA5 as non-self (8). Thus, the correct metabolism of cellular nucleic acids proved critical for the silence of IFN-I signaling under non-infectious conditions (9).

A mounting body of evidence demonstrated the emerging roles of non-coding RNAs (ncRNAs) as regulators of IFN-I signaling. Among the ncRNAs, transcripts having a length of >200 base pair (bp), normally termed as long ncRNAs (lncRNAs), are highlighted in regulating IFN-I signaling in recent years. In this review, we will focus on nucleic acid-mediated IFN-I signaling and summarize progress on the current understanding of lncRNAs regulating the IFN-I signaling pathway.

## IFN-I Signaling Pathway

The activation of IFN-I signaling is initiated by the cell-encoded pattern recognition receptors (PRRs) in response to different kinds of “non-self” stimuli called pathogen-associated molecular patterns (PAMPs) or damage-associated molecular patterns (DAMPs). Although the origins are different, they are equally sensed when exposed to PRRs. Each PRR has its specific subcellular location and substrate: in the endosome, the TLR3 senses dsRNAs, TLR7 and TLR8 senses single-stranded RNAs (ssRNAs), TLR9 binds to DNA (10). In the cytoplasm, RIG-I and MDA5 recognize dsRNAs (11), cyclic GMP–AMP synthase (cGAS) binds to DNA (12). Different PRR-PAMP binding activates different downstream adaptor proteins and then triggers similar cascades for IFN gene expression. For example, cGAS produces cyclic GMP-AMP (cGAMP) to activate stimulator of interferon genes (STING) for downstream cascade (13). On recognizing respective dsRNA substrates, RIG-I and MDA5 both form protein filament on dsRNAs and use mitochondrial antiviral-signaling protein (MAVS) as the adaptor (14). TLR3 recruits TIR-domain-containing adapter-inducing interferon-β (TRIF) while the rest of TLRs associate with myeloid differentiation primary response 88 (MyD88) for IFN signaling activation (15). Adaptors mediated interferon regulatory factor 3 (IRF3) or IRF7 phosphorylation *via* recruiting kinases like TANK binding kinase 1 (TBK1) and NF-κB essential modulator/IκB kinases α and β (NEMO/IKKα/IKKβ). The phosphorylated IRF3 or IRF7 then dimerize and translocate into the nucleus and act as transcription factors for type-I IFN production, such as IFN-α and IFN-β (16). Synergistic actions with other proteins such as p300 and cyclic AMP response element-binding protein (p300/CBP), c-jun/ATF-2 are also required (17, 18).

**Figure 1:**
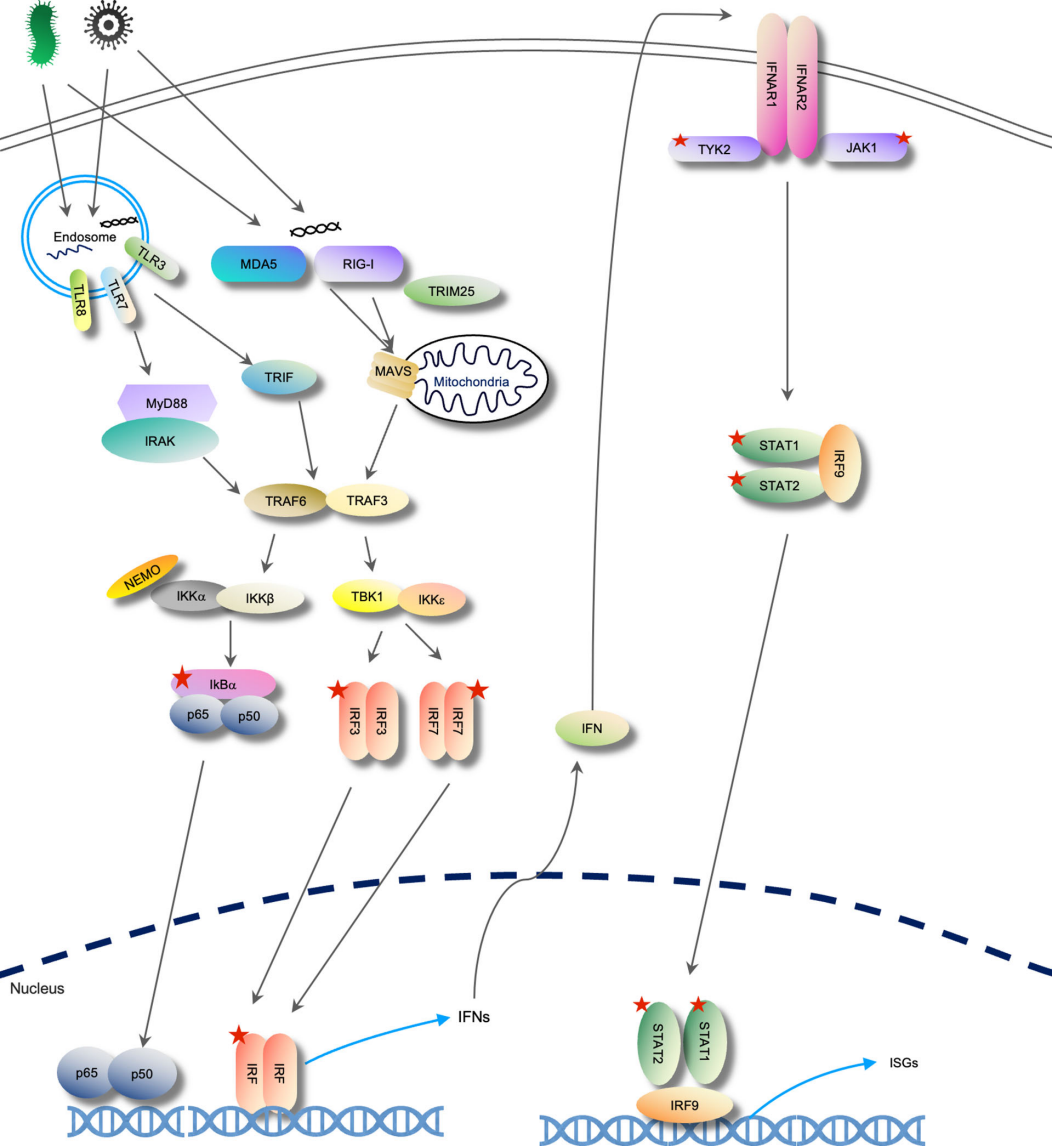
RNA-mediated IFN-I signaling pathway. Pathogen invasion introduces RNA into the endosome or dsRNAs in the cytoplasm. Receptors recognize the RNA molecules and trigger the activation of downstream cascades through their adaptors. IFN-I expression is induced and secreted to activate the ISG expression through IFN-I receptors. Five-sided star represents phosphorylation modification.

Secreted IFNs, such as IFN-α binds to the IFN-α/β receptor (IFNAR) composed of subunits IFNAR1 and IFNAR2 (19). Upon binding, these receptors recruit the Janus kinase (JAK). These tyrosine kinases phosphate each other to get activation and phosphorylates signal transduction factors and activators of transcription (STATs) in the cytoplasm. STAT1, STAT2 is associated with transcription factor IRF9 to form a trimeric complex called IFN-stimulated gene factor 3 (ISGF3). It translocates to the nucleus, binds to genes containing IFN stimulatory response elements (ISREs), and induces transcription of IFN-stimulated genes (ISGs), such as tripartite motif-containing 5 alpha isoenzyme (TRIM5α), oligoadenylate synthetases (OASes), and Mx GTPase family (20, 21). A schematic view of the IFN-I signaling pathway is illustrated in **Figure 1**.

FN signaling pathway is regulated by multiple mechanisms. Positive feedback to strengthen cellular defense and negative feedback to avoid uncontrolled response are both required. Many of the PRRs (e.g., MDA5, RIG-I, TLRs) are themselves ISGs (22). Negative regulators not only play a role in downregulating the activated IFN signaling but also function to maintain the homeostasis of the cellular environment to keep IFN silenced under normal conditions. These feedback players are mostly proteins, either stimulated in expression levels by IFN or constitutively expressed.

Studies in recent decades uncovered important roles of lncRNAs in the IFN signaling pathways. Below, we will introduce the biology of lncRNA and examples of lncRNAs regulating the IFN-I signaling pathway.

## General Biology of LncRNA

About 75% of the human genome can be transcribed (23). However, only 1% of the genome are exons encoding proteins. The majority of the rest transcribed regions produce ncRNAs (24). The ncRNAs can be linear or circular transcripts (25), and function either in a sequence-specific manner (such as small interfering RNA, siRNA, and microRNA, miRNA, albeit their maturation requires certain structures too) or additionally requiring a rigid structure to support (such as tRNAs and rRNAs) (26). Proteins are needed in all cases for proper functions.

LncRNAs are normally defined as non-translating transcripts at a length of >200 bp and are transcribed by RNA polymerase II (Pol II), Pol I, or Pol III (27, 28). Thus, like mRNAs, many Pol II-transcribed lncRNAs are 5′-capped and 3′-polyadenylated (29). LncRNAs can be expressed ubiquitously, such as the paraspeckle component lncRNA NEAT1 (30), or tissue- or cell status-specific, e.g., lncRNA Tcam1 is restricted to express in testicular germ cells (31), as well as several IFN-induced lncRNAs discussed below. Despite being featured as non-coding molecules, evidence suggested that some encode short peptides that play important roles in biological events (32). These peptide-encoding lncRNAs play dual roles through both peptides and RNA and are known as bifunctional RNAs. For example, lncRNA HOXB-AS3 inhibits colon cancer (cancer cell colony formation, proliferation, migration, and invasion) through encoding a 53 amino acids (aa) peptide but not lncRNA itself (33). In endometrial carcinoma (EC) cells, this lncRNA binds to the tumor-suppressing miR-598-5p through sequence annealing and downregulates its level, which in turn, leads to enhanced cancer cell proliferation (34).

Since its first discovery in 2002 by Okazaki et al., more and more lncRNAs have been identified and studied. It was estimated that 58,648 genes encode lncRNAs in the human genome (35). Now we know that lncRNAs participate in almost all aspects of cellular activities, playing important roles to the cells.

Typically, lncRNAs having ~5 kilobases (kb) to the nearest genes are termed intergenic lncRNAs, whose transcriptions are supposed to be more independent of those of neighboring genes. LncRNAs with a protein-coding gene in < 5 kb can be further divided into sense and antisense ones with respect to the neighboring genes (36). Transcription of antisense lncRNAs can be facilitated by the transcription of nearby genes, possibly by the opening of closed double-stranded structure (37, 38), while that of sense lncRNAs can be either unrelated to the nearby genes or even competing with them for transcription (39). Besides, lncRNAs can be derived from intron or exon processing, in which case the terminuses are not capped or tailed (40).

The structure of lncRNAs is established primarily on duplexes due to base pairing and flanked loops and/or arms made of single strands. Although the duplexes are stable, single strands confer plasticity so that the whole conformation can be less rigid than that of protein. Sequences and structures are both important to the biological function of lncRNAs. Because of their sequence diversity and structural flexibility, they participate in multiple cellular events through a variety of mechanisms.

LncRNAs are involved in the regulation of gene expression through three levels: transcriptional regulation, epistasis modification, and post-transcriptional regulation (41). Additionally, lncRNAs are involved in the regulation of various processes in the nucleus and cytoplasm, such as the regulation of immune response, cancer, and stress development, as well as serving as key regulators of cell proliferation and apoptosis (42). It has been shown that neuronal tissues specifically express lncRNAs which are closely associated with neurological disorders, such as Alzheimer’s disease, schizophrenia, autism, and bipolar disorder (43). By participating in the regulation of protein-coding genes, lncRNAs can affect intracellular signaling pathways and also play an important role in embryogenesis and organ differentiation (44). The role of lncRNAs in cancer is extensively studied. Cancer development and progression affected by lncRNAs can be mediated by multiple mechanisms, mainly through epigenetic regulation, activation of carcinogenic pathways, and interference of other RNA isoforms (45). In addition, lncRNAs enhance host defense against invading pathogens in both innate and adaptive immune responses. The role of lncRNAs in the regulation of interferon in innate immunity is described in detail below.

To our best knowledge, the study on the crosstalk between lncRNAs is still limited. Yet, there are complicated associations between lncRNAs and different ncRNAs. For example, the binding of miRNAs to lncRNAs through miRNA response factors (MREs) leads to the decay of bound lncRNAs (46). The interaction also performs a necessary function in gene regulation. Some lncRNAs encode miRNAs and small nucleolar RNAs (snoRNAs) and can also alter the expression of these RNAs (47). Their interactions in the regulation of organism activities have been pronounced successively. In neurodegenerative disorders, interactions between miRNAs and lncRNAs have been mentioned. For example, the beta-site amyloid precursor protein cleaving enzyme 1 (BACE1) is accumulated in Alzheimer’s disease (AD) patients and is studied as a therapeutic target (48). Its antisense transcript, lncRNA BACE1-AS, was found upregulated in brain samples from AD patients. Bioinformatics prediction suggested that miR-485-5p was suggested to target *BACE1* mRNA to downregulate its protein level. It was further found that lncRNA BACE1-AS competes with miR-485-5p on binding onto BACE1 mRNA to restore its translation (49). LncRNAs and miRNAs are enormously related to cancers progression. For example, the miR-200 family represses epithelial-mesenchymal transition (EMT) by binding to the 3′-UTR of *ZEB1* and *ZEB2* mRNA. The TGF-β-induced lncRNA-ATB binds to miR-200s thus restoring the expression of ZEB1 and ZEB2 (50). In addition, MiR-9 has been reported to target lncRNA Malat1 in the nucleus through its binding site and promote tumor cell proliferation and metastasis (51). Interestingly, Malat1 has been proven to play a negative regulatory function in the IFN signaling pathway (will be discussed below), suggesting that the association between different ncRNAs may also play a regulatory role in the immune response. LncRNAs regulating mRNA-targeting miRNAs have impacts on targeted mRNA translation (will be discussed below). A comprehensive review of lncRNA biology can be found elsewhere (38, 46).

## LncRNAs Regulating IFN Signaling

**Figure 2:**
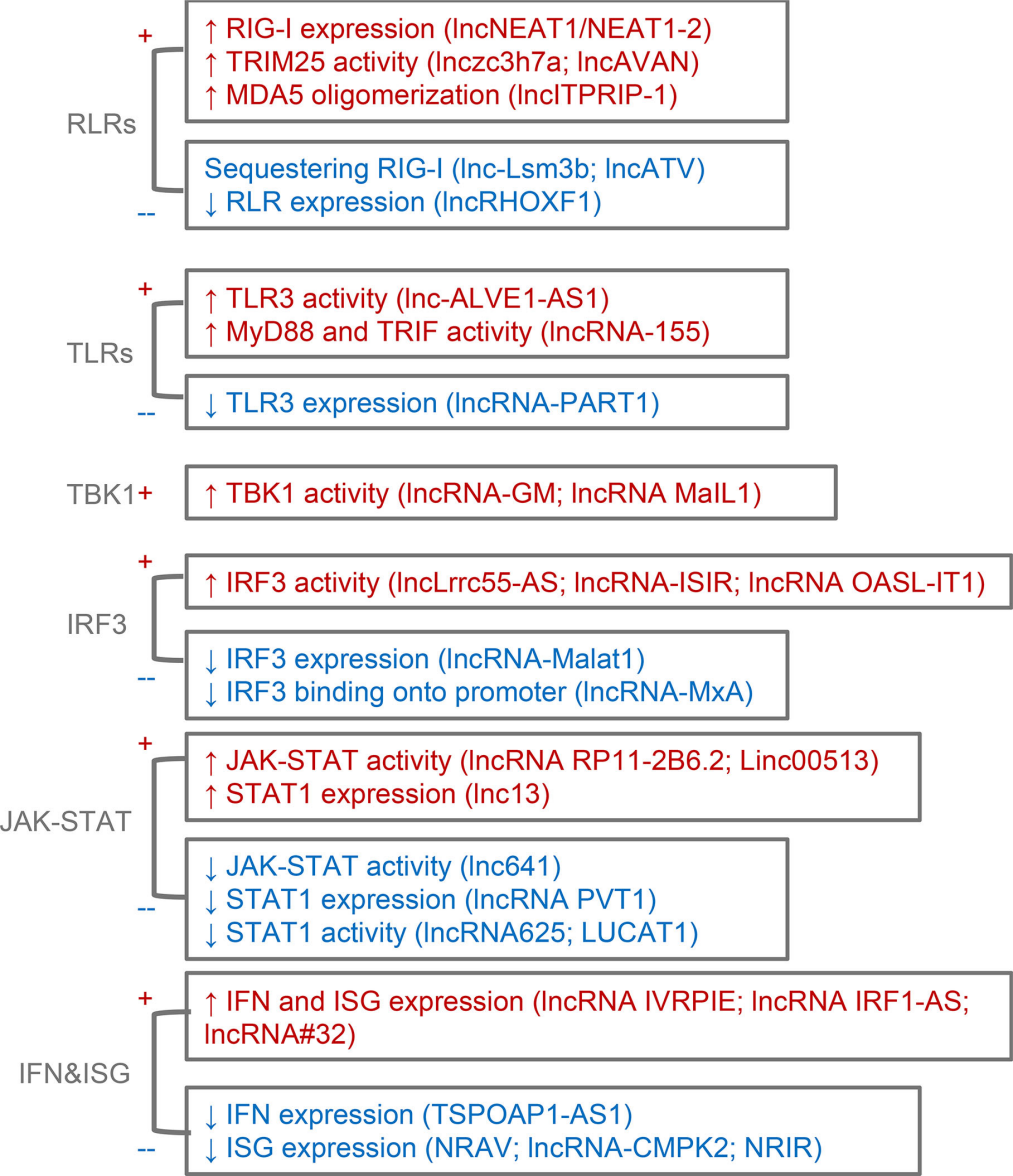
Brief summary of lncRNAs regulating IFN signaling. Based on proteins and events order, lncRNAs affecting RLRs, TLRs, TBK1, IRF3, JAK-STAT axis, IFN, and ISG expression are discussed. +, positive regulation. -, negative regulation.

LncRNAs play important roles in innate and adaptive immune responses. During the IFN signaling, they regulate its activation and function. We summarize this part according to the kind of PRRs and event order during the signaling (**Figure 2**). LncRNAs modulating cellular metabolism other than IFN signaling to influence virus infection, such as lncRNA-ACOD1 (52), are not discussed here. Excellent reviews on this topic can be found elsewhere (38).

### RLR-Targeting lncRNAs

The RIG-I-like receptors (RLRs) include RIG-I, MDA5, and LGP2. All proteins have the helicase domain and C-terminal domain (CTD) for dsRNA recognition and binding. RIG-I and MDA5 additionally are equipped by the N-terminal tandem caspase activation and recruitment domains (CARD) motifs (2CARD in short) for signaling transduction. Thus, albeit all RLRs encode dsRNA-binding capacity, LGP2 is unable to activate IFN-I induction, rather, it acts to modulate RIG-I and MDA5 activity in cells (53). LncRNAs regulate RLR-mediated IFN signaling by regulating multiple aspects, from the RLRs’ expression to protein-protein interactions.

Positive regulatory lncRNAs include NEAT1, lnczc3h7a, AVAN, and lncITPRIP-1. LncRNA NEAT1, nuclear paraspeckle assembly transcript 1, is an essential architectural constituent of paraspeckles in the mammalian nucleus, interacting with proteins such as the splicing factor proline- and glutamine-rich protein (SFPQ) (54). NEAT1 may remove SFPQ from potential promoter regions in the nucleus, which in turn, eliminates SFPQ’s transcriptional repressive activity (54). Its longer transcript variant, NEAT1-2, was identified as an upregulated gene upon a negative ssRNA virus, hantaan virus (HTNV) infection in human umbilical vein endothelial cells (HUVEC) (55). Interestingly, this upregulation during HTNV infection was dependent on activities of RIG-I and IRF7 but NEAT1 itself is not an ISG (55). The increased expression level of NEAT1-2 activates RIG-I-mediated IFN signaling in the context of HTNV infection, possibly through enhancing RIG-I expression level by removing SFPQ from the *RIG-I* promoter (55). Thus, NEAT1-2 serves as a positive feedback regulator of RIG-I-mediated IFN signaling.

RIG-I needs to be K63-linked ubiquitinated for IFN signaling and such post-translational modification relies on E3 ligases such as ring finger protein 135 (RNF135, also known as RIPLET) and tripartite motif-containing protein 25 (TRIM25) (56). Interestingly, TRIM25 itself is targeted and regulated in function by lncRNAs, such as lnczc3h7a and lncRNA AVAN. Lnczc3h7a was identified as a TRIM25-interacting lncRNA in murine macrophage RAW264.7 cells upon vesicular stomatitis virus (VSV) infection. It has a length of 603 nucleotides (nt) with a poly (A) tail at the 3′-end and is suggested to be upregulated in expression in an IFN-I-dependent manner (57). It promotes RIG-I-dependent, but not MDA5 or cGAS-dependent antiviral response against RNA viruses infection. Its knockdown significantly reduced the induction of IFN-I, ISGs, and pro-inflammatory cytokines in peritoneal macrophages infected with VSV or Sendai virus (SeV) (recognized by RIG-I) but had no effect when infected with encephalomyocarditis virus (EMCV) (recognized by MDA5), herpes simplex virus -1 (HSV-1) (recognized by cGAS) or treated with LPS (recognized by TLR4) (57). Mechanism study indicated that lnczc3h7a promotes RIG-I oligomerization by facilitating the interaction between RIG-I and TRIM25, an E3 ligase mediating the K63 linked ubiquitination of RIG-I for its activation. Lnczc3h7a acts as a scaffold through binding to the helicase domain of filamentous RIG-I and SPRY domain of TRIM25 in the cytoplasm (57). AVAN is another TRIM25-targeting lncRNA in the cytoplasm, having a length of 517 nt (58). It is upregulated in expression upon influenza A virus (IAV) infection to human neutrophils and is required for the antiviral response against IAV infection. Sequence alignment analysis suggested that it is not conserved between humans and mice. Interestingly, forced expression of this lncRNA in mice significantly inhibits IAV infection. Immunoprecipitation assays showed that it binds directly to the B-Box/coiled-coil motifs of TRIM25 to enhance TRIM25 activity on mediating K63-linked RIG-I ubiquitination and this interaction is also seen with rodent TRIM25, explaining why AVAN functions in mice (58). It was suggested that AVAN is slightly upregulated in expression by multiple RNA viruses (e.g., SeV), poly I:C, and interferon treatment, but not adenovirus (ADV).

Both RIG-I and MDA5 need to form respective filamentous protein-protein oligomers or multiplexers along the length of dsRNA, for 2CARD oligomerization and activating MAVS subsequently (59). This process can also be regulated by lncRNAs. LncITPRIP-1 was identified to be stimulated by IFN-α treatment in human hepatocytes during RNA virus (e.g., VSV and SeV) and DNA virus (e.g., HSV) infection (60). Data showed that lncITPRIP-1 restricts hepatitis C virus (HCV) infection through enhancing HCV-triggered IRF3 activation. RNA immunoprecipitation (RIP) assay suggested that lncITPRIP-1 promotes MDA5 oligomerization possibly through binding to the C-terminus of MDA5, albeit it also enhanced 2CARD alone oligomerization independent of RNA-protein interaction. Such mode of action enables lncITPRIP-1 to strengthen MDA5 filament formation along the length of HCV dsRNA (60).

Besides these positive regulatory lncRNAs, RLR activity is also modulated negatively by some types of lncRNAs. The mouse genome encoded Lnc-Lsm3b was identified as a RIG-I binding lncRNA following VSV infection in mouse macrophage RAW264.7 cells. Further study showed that this lncRNA was upregulated in expression upon RNA virus (e.g., VSV and SeV) infection or 5′-ppp dsRNA transfection, and negatively regulates RIG-I mediated IFN signaling (61). Its sequence is embedded in the *Lsm3b* gene and is transcribed from the *Lsm3* loci in response to viral infection (61). Lnc-Lsm3b binds to RIG-I to sequester it from forming oligomers. Thus, it competes with dsRNA substrate to reduce RIG-I activity (61). This mode of action is supposed to facilitate RIG-I keeping inactive in the non-infected stage. Another RIG-I-specific negative regulatory lncRNA is the human-specific lncATV, which is upregulated by IFN-I, IFN-III, or virus (VSV, HCV, SeV) infection in Huh7 cells. Its knockdown inhibits the replication of HCV, Zika virus, NDV, and SeV infection. LncATV exerts its role by interacting with the full-length RIG-I (62).

LncRHOXF1 is another negative regulator of RLRs. It was identified in a survey of highly expressed lncRNAs in trophectoderm but not the inner cell mass (63). Silencing of *lncRHOXF1* by siRNA protects early human trophoblast progenitor cells from SeV infection and an enhanced ISG expression was observed, including the dsRNA cytosolic sensors RIG-I and MDA5. SeV infection increases this lncRNA expression, implicating this lncRNA is either an ISG or directly upregulated by virus infection. This phenomenon is restricted to trophoblast progenitor cells (63) and the mechanism for its regulatory role on virus response needs further investigation.

**Figure 3:**
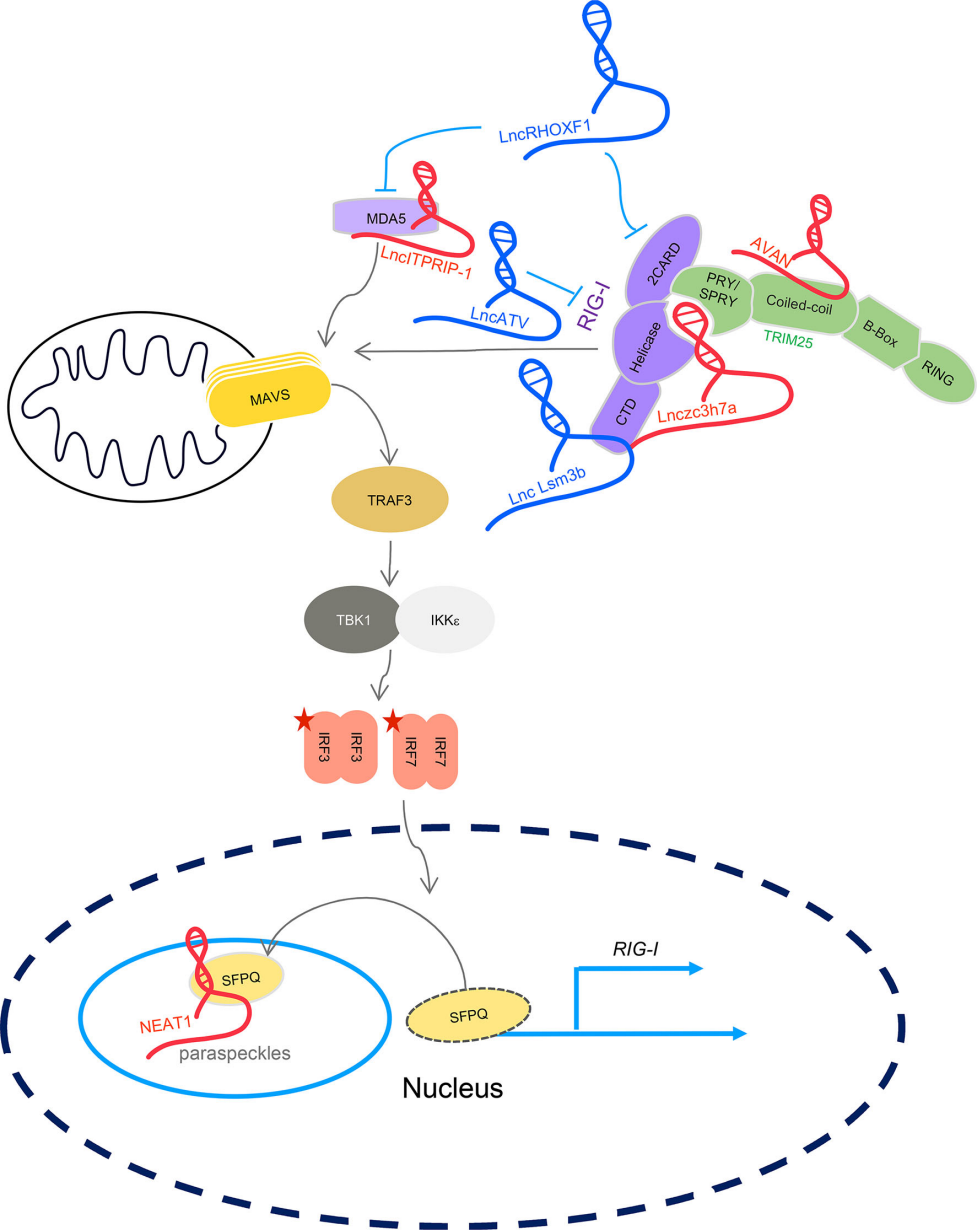
LncRNAs regulates RLR-mediated IFN signaling. LncRNA NEAT1, lnczc3h7a, AVAN, and lncITPRIP-1 positively regulate IFN signaling through targeting proteins indicated in the figure. Lnc-Lsm3b, lncATV, and lncRHOXF1 negatively regulate IFN signaling by targeting RIG-I and RLRs. Five-sided star represents phosphorylation modification. LncRNA in blue represents negative regulators; lncRNA in red is positive regulators for IFN signaling.

As can be seen, lncRNAs encode both positive and negative regulatory activities to guarantee a correct IFN-I signaling activation by RLRs. A schematic view of lncRNAs regulating RLRs-mediated IFN signaling is illustrated in **Figure 3**.

### TLR-Targeting lncRNAs

TLRs play critical roles for the host to defend against pathogen invasion. Unlike RLRs localize in the cytoplasm, where it is easier for lncRNAs to access and bind, TLRs are physically barriered by the lipid membranes. Furthermore, endosome-presented lncRNAs may themselves be recognized as ligand to stimulate local TLRs. Thus, lncRNAs working on TLRs might either act as ligands themselves (lnc-ALVE1-AS1) or regulators targeting TLR adaptors in the cytosol (LncRNA-155, MaIL1, and PART1).

Lnc-ALVE1-AS1 was identified from an RNA-seq of 5-Aza-2′-deoxycytidine (5-Aza-CdR)-treated chicken embryo fibroblast (CEF). It is in the endogenous retrovirus (ERV) *ALVE1* locus and derived from its antisense transcription, having a length of 2135 nt (64). 5-Aza-CdR is a DNA methylation inhibitor and is used in cancer therapy. Data showed that it functions to kill cancer cells dependent on the MDA5-mediated IFN-I signaling. 5-Aza-CdR induces the transcription of ERVs on both strands, therefore, dsRNAs are produced and sensed by MDA5 (65). Lnc-ALVE1-AS1 mainly localizes in the cytoplasm and its expression activates IFN signaling. Further study found that it co-localizes with TLR3 in CEFs, possibly serving as a ligand to activate TLR3 (64). Considering the length of this lncRNA, duplex structures are likely formed intramolecularly. However, how lnc-ALVE1-AS1 enters the endosome is still unknown. It is also interesting to investigate if this sequence is conserved among humans, mice, and other mammal species.

LncRNA-155 expression is stimulated by IFN-β treatment and is induced by IAV and other RNA viruses (e.g., Muscovy duck reovirus and SeV) infection, dependent on RIG-I and TLR3, in human lung carcinoma epithelial cell line, A549 (66). It localizes in both the nucleus and cytoplasm, albeit more is accumulated in the nucleus. It acts as positive feedback to IFN signaling by reducing the expression of protein tyrosine phosphatase-1B (PTP1B), a protein negatively regulates MyD88 and TRIF-dependent pro-inflammatory cytokine induction and IFN-I production (66, 67). Nevertheless, the mechanism by which lncRNA-155 suppresses PTP1B remains to be elucidated.

Not all relevant lncRNAs enhance TLR activities. LncRNA-PART1 was identified in a survey of differentially expressed lncRNAs in prostate cancer (PC) tissue samples compared to adjacent tissues, where it was highly expressed in PC (68). It facilitates PC cell proliferation and its knockdown triggers apoptosis. Cellular assay further found that this lncRNA negatively regulates the expression level of *TLR3, TNFSF10*, and *CXCL13* (68), all of which were reported to be related to apoptosis (69, 70).

**Figure 4:**
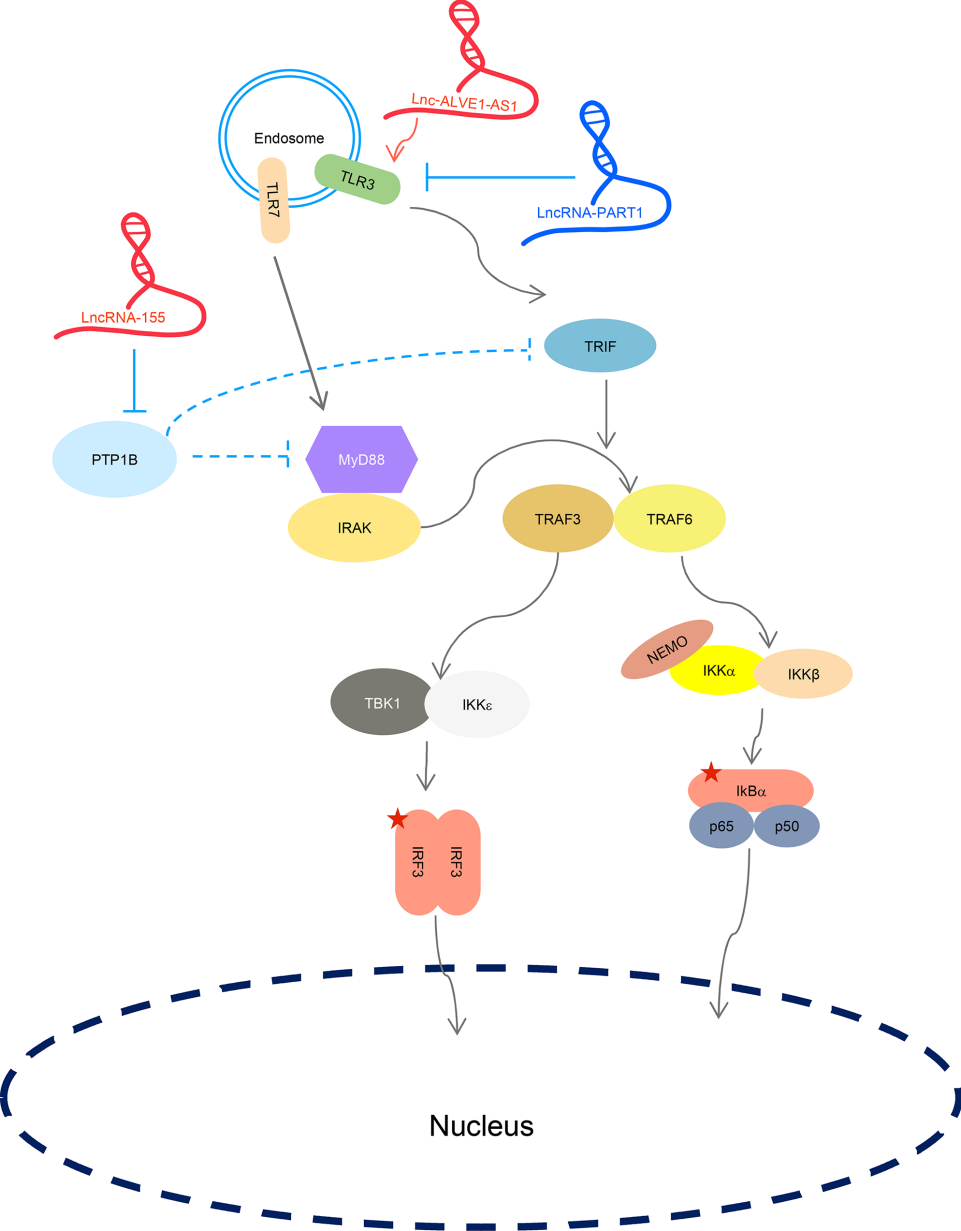
LncRNAs regulate TLR-mediated IFN signaling. LncRNA ALVE1-AS1 enhances TLR3-mediated IFN signaling. LncRNA-155 targets PTP1B. LncRNA-PART1 negatively affects TLR-mediated IFN signaling. “U” represents ubiquitination modification. Five-sided star represents phosphorylation modification. LncRNA in blue represents negative regulators; lncRNA in red is positive regulators for IFN signaling.

Different lncRNAs play different roles in regulating endosomal TLR activity. The effects of LncRNAs on TLR-mediated IFN signaling are described in **Figure 4**.

### General Downstream Cascades for IFN-I Transcription Activation

One of the features of IFN-I signaling activation is that despite different ligands being recognized by different receptors, followed by activating respective adaptors, their downstream events overlap, such as kinases activation, IRF3, or IRF7 phosphorylation, and dimerization. The following section will discuss lncRNAs regulating these downstream cascades, from TBK1 to IRF3 and NF-κB.

TBK1 is a critical kinase in IFN signaling activation. Studies showed that lncRNAs such as lncRNA-GM and MaIL1 promote TBK1 activity. LncRNA-GM was identified through functional screening in mouse embryonic fibroblasts after VSV infection. And downregulation of lncRNA-GM caused by RNA virus (such as VSV and SeV) or DNA virus (e.g., HSV-1 and vaccinia virus) is dependent on the IRF3-IFN-I pathway. LncRNA-GM locates in the cytoplasm. It regulates IFN-I production through improving TBK1 phosphorylation (71). Glutathione S-transferase mu 1 (GSTM1) mediates the glutathionylation of TBK1 and such modification inhibits TBK1 phosphorylation. LncRNA-GM binds to GSTM1 to sequester it and rescue TBK1 activity (72). Upon RNA viruses (such as VSV and SeV) and DNA viruses (e.g., HSV-1, vaccinia virus) infection, lncRNA-GM is downregulated in expression through a not-yet understood mechanism and thus frees GSTM1, which in turn, mediates the glutathionylation of TBK1 at the Cys637 site to reduce TBK1 kinase activity (72). Another TBK-targeting lncRNA is lncRNA MaIL1. It is upregulated in expression during macrophage activation by LPS treatment and positively regulates TLR4 activity by binding to optineurin (OPTN) (73). OPTN is a ubiquitin-adaptor and aggregates to platform TBK1 phosphorylating IRF3 (74). The OPTN-MaIL1 binding stabilizes OPTN and is required for OPTN-TBK1 activation. Interestingly, silencing of MaIL1 by siRNAs has a more profound impact on LPS-directed IFN signaling than on poly I:C-dependent IFN signaling (73), suggesting a preference of MaIL1 on TLR4-mediated IFN signaling.

The NEMO/IKKα/IKKβ complex is another critical player in signaling transduction. It was demonstrated that in macrophages, TRIM29 mediates NEMO degradation through directly binding to NEMO, inducing its ubiquitination and thus proteolytic degradation. Such a negative regulatory role avoids the lethal effect on mice upon *Haemophilus influenzae* infection because deletion of *Trim29* in mice caused host death due to overproduction of proinflammatory cytokines by macrophages (75). TRIM29 is also stimulated in expression upon poly I:C transfection in human myeloid dendritic cells (mDCs) and then interacts with MAVS to induce MAVS’s K11-linked ubiquitination and degradation subsequently (76). TRIM29 deficiency protects the mice from reovirus T3D strain infection, a lethal dsRNA virus to the wild-type mice, due to the enhanced antiviral activity. Studies of papillary thyroid cancer (PTC) suggested that TRIM29 may be regulated by lncRNA indirectly. TRIM29 contributes to PTC cell proliferation (77). miR-761 targets the 3′-UTR of *TRIM29* mRNA to downregulate TRIM29 expression and thus inhibits its biological role in PTC cells. In the same study, the miR-761 level was found downregulated in PTC. Starbase v2.0 predicted that lncRNA HOXA11-AS may target and lower the level of miR-761. Expression tests confirmed such correlation and indicated that lncRNA HOXA11-AS promotes PTC cell proliferation (77). Another group also found the beneficial role of TRIM29 in PTC cell proliferation, whose mRNA can be targeted by miR-195-5p (78). They observed that another lncRNA, 00324 (LINC00324), rescues TRIM29 expression by targeting miR-195-5p (78). Whether lncRNA HOXA11-AS and LINC00324 affect IFN signaling through regulating TRIM29 expression is of interest to know.

IRF3 is the key transcription factor for IFN-I transcription. It is phosphorylated in the cytosol and dimerized afterward. The IRF3 dimer then translocates to the nucleus to bind to the IFN-I promoter for transcription activation. LncLrrc55-AS acts as a positive regulator of IRF3 activity. It is derived from the antisense transcripts of the Lrrc55 (leucine-rich repeat containing 55) gene, which has 286 nt with a 3′-polyadenylated tail and locates in the cytoplasm. LncLrrc55-AS is upregulated upon VSV infection in macrophages. The upregulation of lncLrrc55-AS can be triggered by other innate stimuli, including DNA virus (e.g., HSV), as well as LPS, poly I: C, and IFN-I (79). It promotes IRF3-mediated IFN-I signaling. Silencing of lncLrrc55-AS in macrophages, NIH/3T3 cells by CRISPR-Cas9 system significantly reduced IRF3 phosphorylation after SeV infection (79). LncLrrc55-AS interacts with phosphatase methylesterase 1 (PME-1) to strengthen the interaction between PME-1 and phosphatase protein phosphatase 2A (PP2A), which in turn, facilitates the demethylation and inactivation of PP2A. Such mode of action promotes IRF3 phosphorylation and the IFN-I signaling (79).

A more straightforward IRF3-targeting lncRNA is LncRNA-ISIR. It directly associates to IRF3, improving its phosphorylation, dimerization, nuclear translocation, and the transcriptional activation of IFN-I (80). It was identified through formaldehyde-crosslinked RNA immunoprecipitation (FA-CLIP) with anti-FLAG antibody enriched knock-in FLAG-tagged IRF3 complex in primary mouse peritoneal macrophages after infection of VSV. The mechanistic study showed that IRF3 is sequestered by a repressor protein called Flightless-1 (Fli-1) in non-infected conditions to avoid aberrant activation. Virus infection leads to IFN-I signaling activation and this lncRNA upregulation, which in turn, binds to IRF3 to remove Fli-1 (80).

LncRNA OASL-IT1 is a new ISG identified by microarray assay in A549 cells infected with Zika virus (ZIKV). LncRNA OASL-IT1 is intronic and distributed in both cytoplasm and nuclei. LncRNA OASL-IT1 positively regulates IFN-I signaling (81). Silencing of its expression *via* knockout or knockdown impairs the phosphorylation modification of p38 of mitogen-activated protein kinases (MAPK), IRF3, and NF-κB p65 in response to ZIKA infection (82). The detail of its mechanism is still not understood.

LncRNA Malat1, on the contrary, is a negative regulator of antiviral type I IFN production. LncRNA Malat1 is in nuclei and expression of which was repressed when VSV infected in n RAW264.7 cells. Expression of lncRNA Malat1 was also reduced when treated EMCV or HSV-1, but not LPS or poly I:C. The transactive response DNA binding protein 43 (TDP43) can be cleaved by caspase 3 into its active form TDP35, which in turn inhibits the expression of E3 ligase of IRF3, Ring-B-box-coiled-coil protein interacting with protein kinase C-1 (Rbck1) through degrading Rbck1 pre-mRNA (83). Rbck1 mediates IRF3 ubiquitination for proteasomal degradation upon viral infection. Thus, the activity of TDP35 positively regulated IRF3-directed IFN signaling. LncRNA Malat1 binds directly to the RRM1 domain of TDP43 in the nucleus to block its cleavage (84). Upon viral infection, Malat1 expression is downregulated and TDP43 is released to become TDP35 (85).

IRF3-promoter binding can also be impaired by lncRNAs. LncRNA-MxA is embedded in the *MxA* locus in the genome and is an ISG too, like *MxA* (86). Different from the *MxA* mRNA, which is transported to the cytoplasm for protein translation, lncRNA-MxA stays in the nucleus in IAV-infected cells. Its ectopic expression in HEK293T cells enhanced IAV replication and its knockdown decreased viral replication. The further assay showed that lnc-MxA functions downstream of IRF3 but does not bind directly to IRF3, as observed in an RIP assay. Rather, it was observed to disrupt the association of IRF3 with the *IFNβ* promoter using a chromatin immunoprecipitation (ChIP) assay. This disruption is done through lnc-MxA binding onto the promoter sequence to form a triple-stranded complex (86). Whether other binding proteins are required in this binding process or not is of interest to know.

**Figure 5:**
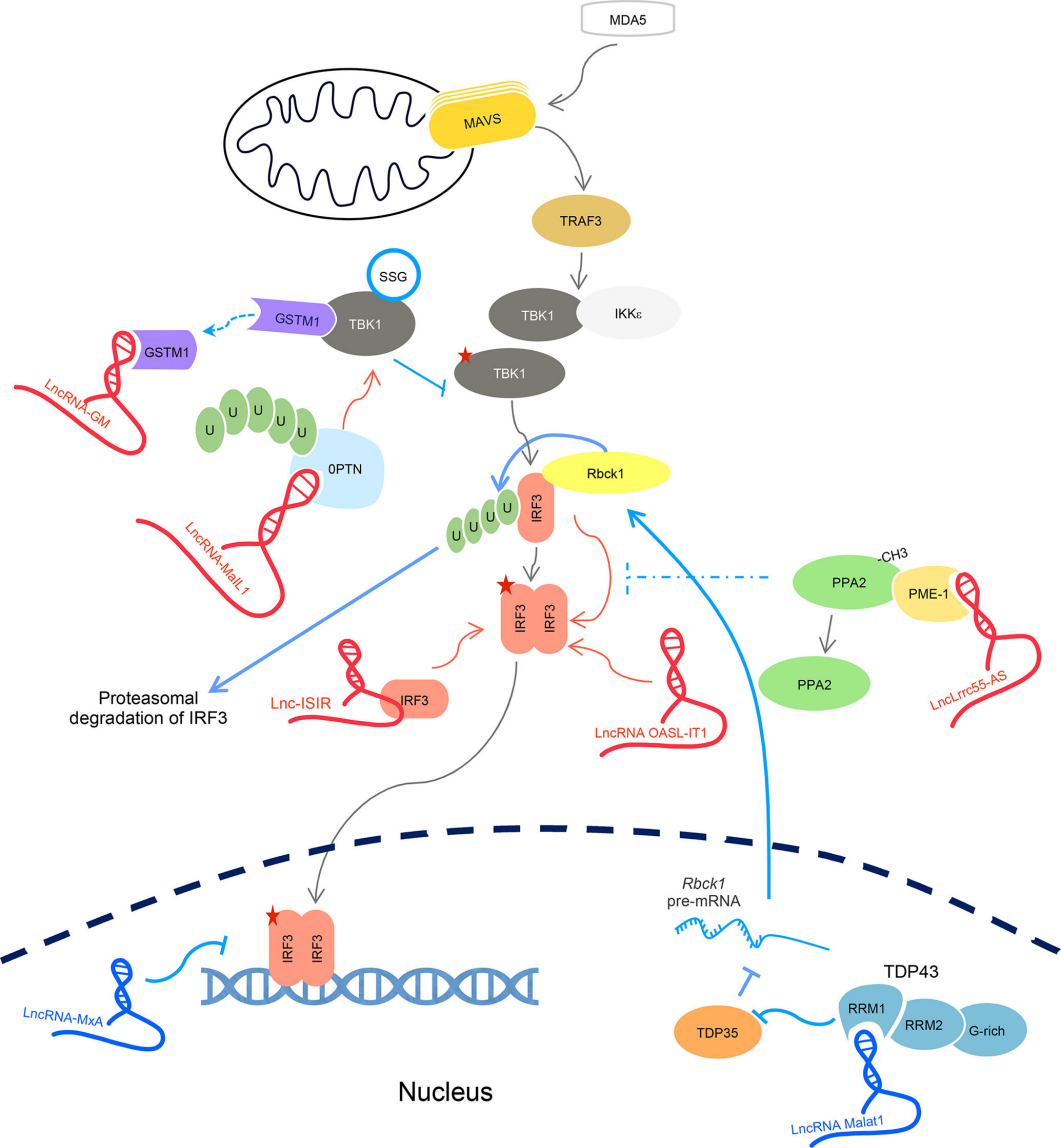
LncRNAs regulate downstream cascades for IFN-I activation. LncRNA-GM targets GSTM1 to disrupt GSTM1-directed S-glutathionylation of TBK1 (SSG), thus promoting TBK1 for its kinase activity. LncRNA MaIL1 targets OPTN to facilitate OPTN-TBK1 binding for TBk1 kinase activity. LncRNA OASL-IT1, lncRNA-ISIR, and lncLrrc55-AS facilitate IRF3 phosphorylation. LncRNA Malat1 downregulates IRF3 abundance by targeting TDP43. LncRNA-MxA inhibits IRF3 binding onto the IFN promoter region. Five-sided star represents phosphorylation modification. LncRNA in blue represents negative regulators; lncRNA in red is positive regulators for IFN signaling.

A summarize is illustrated in **Figure 5**.

### The JAK-STAT Axis

IFN is secreted to bind to receptors on self and neighboring cells. Some lncRNAs act after IFN production by affecting IFN autocrine or paracrine-induced downstream signaling, such as the JAK-STAT cascade. LncRNA RP11-2B6.2 is a positive regulator of this axis (87). It was identified as an upregulated lncRNA and positively correlated with the expression of ISGs through performing next-generation RNA sequencing in the kidney tissues and peripheral blood mononuclear cells (PBMCs) of lupus nephritis (LN) patients. Knockdown of lncRNA RP11-2B6.2 in renal cells suppresses the expression of ISGs, while its overexpression enhances ISGs’ expression. Evidence suggested that it reduces the expression of suppressor of cytokine signaling 1 (SOCS1), a protein negatively regulates the phosphorylation of JAK1, tyrosine kinase 2 (TYK2), and STAT1 in the IFN-I pathway (88).

Linc00513 was identified as a positive regulator of IFN-I signaling in a transcriptome survey of renal tissues of systemic lupus erythematosus (SLE) susceptibility loci (89). Linc00513 locates in the nucleus showing punctate aggregation distribution and is partially dispersed in the cytoplasm. It promotes the phosphorylation of STAT1 and STAT2 and is expressed at high levels in patents with active disease (89).

Another STAT1 expression-enhancing lncRNA is lnc13. It is a heterogeneous nuclear ribonucleoprotein (hnRNP)-binding lncRNA that binds to the p42 isoform of hnRNPD and histone deacetylase 1 (HDAC1), a component of the nucleosome remodeling and deacetylating (NuRD) remodeling complex (90). The expression level of this lncRNA is significantly upregulated in pancreatic β-cells challenged by a diabetogenic Coxsackie Virus B5 (CVB5) or intracellular poly I:C, but not by IL-1β or IFN-γ. These stimuli induce lnc13 translocation from the nucleus to the cytoplasm promoting the interaction of *STAT1* mRNA with (poly[rC] binding protein 2) (PCBP2), which in turn stabilize *STAT1* mRNA and enhance protein translation. It functions during the inflammatory signaling, recruiting the chromatin modification enzyme HDAC1 to change the chromatin structure, deacetylating histones to restrict pro-inflammatory gene transcription (90). hnRNPD is required for lnc13 binding onto HDAC1 and the subsequent inhibition of transcription (90), implicating its bridging role during this process.

Lnc641, on the contrary, suppresses the innate immune response to pseudorabies virus (PRV) infection in porcine monomyeloid cell line, 3D4/21, by downregulating IFN-α production through the JAK/STAT pathway. It reduces the phosphorylation of JAK and STAT1 to inhibit ISG expression (91). Yet, how lnc641 regulates the changes of JAK/STAT1 and IFN needs further research.

LncRNA PVT1 also negatively affects IFN signaling. Its expression level was significantly increased in hepatocellular carcinoma cells than in the normal hepatocyte cells. This difference may connect to low methylation of CpG islands, an aberrant DNA methylation, in *PVT1* promoter regions. The study further found that lncRNA PVT1 was upregulated after IFN-α treatment. Only full-length lncRNA PVT1 interacts with STAT1, whereas the truncated form does not. Its knocking down enhances the expression level of STAT1 and nuclear translocation of phosphorylated STAT1 and STAT2 (92).

LncRNA625 was identified through its high expression in esophageal squamous cell carcinoma (ESCC) (93). LncRNA625 was predominantly localized in the nucleus by RNA fluorescence *in situ* hybridization (FISH) and cell fractionation assays. It is possibly not an ISG because IFN-γ treatment does not alter the expression of lncRNA625. Mechanistically, lncRNA625 acts through interacting with the DNA-binding domain of STAT1, which in turn, promotes the interaction of STAT1 with the T-cell protein tyrosine phosphatase, TC45 to dephosphorylate STAT1, thereby inhibiting STAT1 activation and preventing ISGs’ transcription in the nucleus (94).

Another STAT1-promoter binding-affecting lncRNA is LUCAT1. LUCAT1 showed a significant increase in expression after stimulation with LPS, HSV-1, and IAV in primary human monocyte-derived dendritic cells (hMDDCs) or monocytic cell lines. It suppresses the expression of inflammatory genes and ISGs during the acute phase of infection. Following induction, LUCAT1 is highly enriched in the nucleus and interacts with STAT1 to reduce STAT1 binding on ISGs’ promoter sites (95).

**Figure 6:**
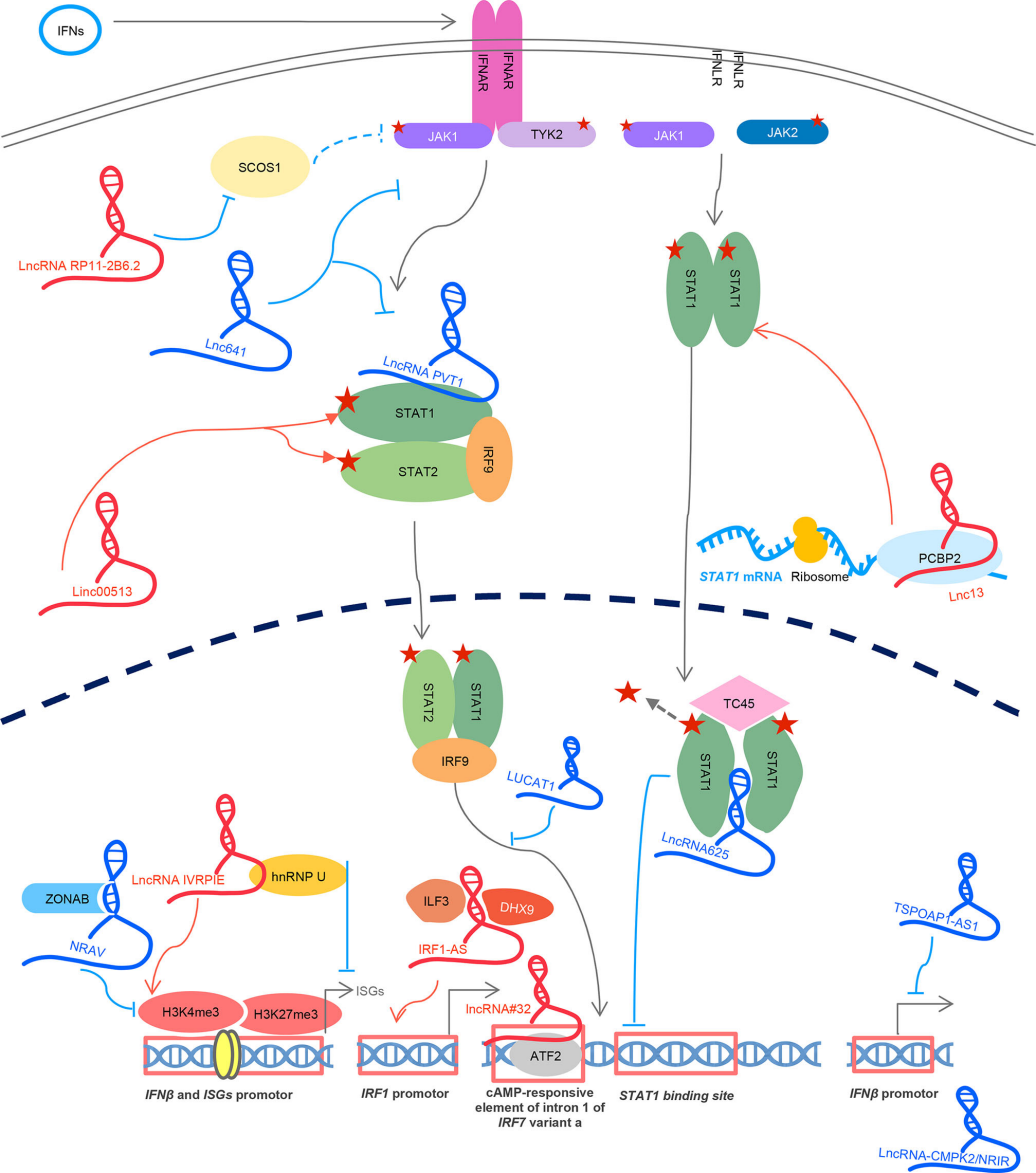
LncRNAs regulates IFN and ISG transcription. IFN signals through binding to the receptors and activation of downstream JAK-STAT cascades. LncRNA RP11-2B6.2, linc00513, and lnc13 enhance JAK-STAT activities. LncRNA IVRPIE, IRF1-AS, and lncRNA#32 enhance IFN and ISG transcription. Lnc641, PVT1, lncRNA625, and LUCAT1 downregulate JAK-STAT activity. LncRNA TSPOAP1-AS1, NRAV, and lncRNA-CMPK2 decrease IFN and ISG transcription. Five-sided star represents phosphorylation modification. LncRNA in blue represents negative regulators; lncRNA in red is positive regulators for IFN signaling.

A summary of lncRNAs mentioned above is illustrated in **Figure 6**.

### Transcription and RNA Stability of IFN-I and ISGs

The production of IFN-I and ISGs relies on active gene transcriptional activities. Chromatin structures have profound roles in modulating transcription. For example, the above-mentioned LUCAT1 also recruits chromatin modification complexes or transcriptional repressors to alter chromatin state and block transcription of STAT1 target genes, such as interferon gamma inducible protein 16 (IFI16) and MxB (95).

LncRNA IVRPIE (Inhibiting IAV Replication by Promoting IFN and ISGs Expression) is preferentially induced in expression by an existing RNA sequencing to define the transcriptome of peripheral blood leucocyte samples from patients infected with IAV (96). IVRPIE is also significantly upregulated by stimuli including SeV, VSV, and poly I:C, but not upregulated by other RNA viruses like respiratory syncytial virus (RSV) or DNA viruses like ADV, in A549 cells and a human bronchial epithelium BEAS-2B cells. It is noted that this inducible expression pattern was not observed in HEK293T, HepG2, or HeLa cells, implicating its role in peripheral blood leucocytes and lung cells. IVRPIE is 1316 nt in length and is mostly located in the nucleus. It modulates chromatin status at the transcription start site (TSS) to facilitate the enrichment of tri-methylation of lysine 4 on histone H3 (H3K4me3) and thus promotes the transcription of IFN-β and ISGs (96).

Another transcription activating lncRNA is lncRNA IRF1-AS. It is an IFN-inducible (IFN-β and IFN-γ *via* the JAK-STAT pathway) nuclear lncRNA. LncRNA IRF1-AS has three exons, and exon 3 has a region overlapping with *IRF1*. It was upregulated in three ESCC cell lines (KYSE30, KYSE180, and KYSE450) after being treated with IFN-β. LncRNA IRF1-AS interacts with interleukin enhancer binding factor 3 (ILF3) and DExH-Box helicase 9 (DHX9) to activate IRF1 transcription. IRF1 in turn binds to the promoter region of lncRNA IRF1-AS to drive this lncRNA transcription (97). Besides, IRF1-AS acts as a tumor suppressor inhibiting tumor growth *in vitro* and *in vivo* (97, 98).

LncRNA#32, also known as lncRNA upregulator of antiviral response interferon signaling (LUARIS), is associated with type I IFN signaling. LncRNA#32 transcript is 2,946 nt in length. It was identified as a downregulated lncRNA in human primary hepatocyte HuS cells stimulated with poly I:C. Treatment with IFN-β also suppressed lncRNA#32 expression in HuS cells. LncRNA#32 positively regulates the host antiviral response. Silencing of lncRNA#32 results in reduced mRNA levels of several ISGs, including *OASL*, *RSAD2*, *IP-10, APOBEC3A*, and *APOBEC3G* (99). It interacts with activating transcription factor 2 (ATF2), which binds to the cAMP response element (CRE) in the first intron region of *IRF7* variant a to promote transcription (99). Additionally, lncRNA#32 binds to hnRNPU and is stabilized by such binding (99).

Again, there are lncRNAs negatively function in the transcription of IFN and ISGs. TSPOAP1-AS1 is upregulated in expression in A549 cells upon IAV infection or poly I:C transfection. Noted that transfection mostly delivers poly I:C to cytoplasm whereas addition into the medium directly would more likely transport poly I:C to endosomes. Thus, it is supposed that poly I:C transfection triggers MDA5- or RIG-I-mediated IFN signaling, instead of TLR3-mediated IFN signaling. It enhances IAV replication through inhibiting IFN-β transcription, consistent with the observation that it localizes in the cytoplasm and nucleus (100). The mechanism needs further investigation.

ISG-impairing lncRNAs include lncRNA NRAV, lncRNA-CMPK2. LncRNA NRAV, whose name stands for the negative regulator of antiviral response, was identified as a lncRNA downregulated in expression in influenza virus-infected human alveolar epithelial cells (A549). It is also negatively impacted in expression in several cell lines infected by SeV, Muscovy Duck reovirus (MDRV) (a dsRNA virus), or HSV. Strikingly, ectopic expression of this lncRNA enhanced IAV infection, and knockdown of it suppressed viral replication (101), implicating NRAV acts to assist viral infection rather than defend against it. Thus, the expression level changes upon viral infection seem to be a cellular self-rescue action rather than a consequence of hijack by viruses. The further study uncovered that NRAV down-regulates the host antiviral response by inhibiting selected histone methylation (H3K4me3 and H3K27me3) of several ISGs, including *IFITM3* and *MxA*, thereby repressing the transcription initiation of these genes (101). Besides, NRAV specifically binds to the multi-functional transcription factor zonula occludens 1-associated nucleic acid binding protein (ZONAB), a transcriptional regulator of cyclin D1 and proliferating cell nuclear antigen (PCNA). ZONAB positively regulates the transcription of *MxA* and can rescue NRAV-mediated transcriptional repression of *MxA*. The mechanism of the inhibitory effect of NRAV binding to ZONAB and how ZONAB regulates the expression of other ISGs requires further experiments to demonstrate (101).

LncRNA-CMPK2, also known as NRIR (negative regulator of interferon response), locates downstream of an ISG, *CMPK2*, in a head-to-tail non-overlapping manner. It is induced in human primary hepatocytes upon IFN-α treatment and is expressed at high levels in HCV patients’ liver tissues (102) and SLE patients (103). Knockdown of this lncRNA leads to reduced replication of HCV and enhanced ISG expression at both basal and stimulated states. Thus, it negatively regulates IFN signaling through inhibiting ISGs’ transcription. In line with this, lncRNA-CMPK2 localizes dominantly in the nucleus (102), implicating it interacts with either chromosol DNA directly or DNA-binding proteins to impact ISG transcription. In hepatocytes and epithelial cells, lncRNA-CMPK2 negatively regulates the expression of selected ISGs (e.g., *CMPK2, CXCL10, IFIT3, IFITM1, ISG15, Viperin*, and *IFITM3*) (102, 104). Because it exists in the nucleus, the mechanism of its regulation is presumed to act at the transcriptional or epigenetic level (105), which needs further study. Contrary to its negative regulation in hepatocytes, lncRNA-CMPK2 positively regulates TLR-mediated activation of interferon signaling pathways in monocytes. It was identified in a survey for responsive lncRNAs upon TLR4 activation by LPS treatment in monocytes (105). Further assays found that its expression is stimulated by activated TLRs (TLR3, TLR4, TLR7, or TLR8) in a manner dependent on IFN-I signaling, as the blockade of IFN-α receptor *via* antibodies abolished LPS-mediated NRIR induction (105). It is also upregulated in macrophages infected by Mycobacterium tuberculosis (106). In human monocytes, the silencing of this lncRNA by siRNAs reduces the expression of TLR3, TLR4, TLR7, or TLR8 mediated IFN-I signaling activation and ISG expression (105), implicating a positive correlation of NRIR expression and ISG signatures. Thus, the role of this lncRNA seems to be different according to cell lines, cell status, and stimuli.

A summary is illustrated also in **Figure 6**.

## Discussion

**Figure 7:**
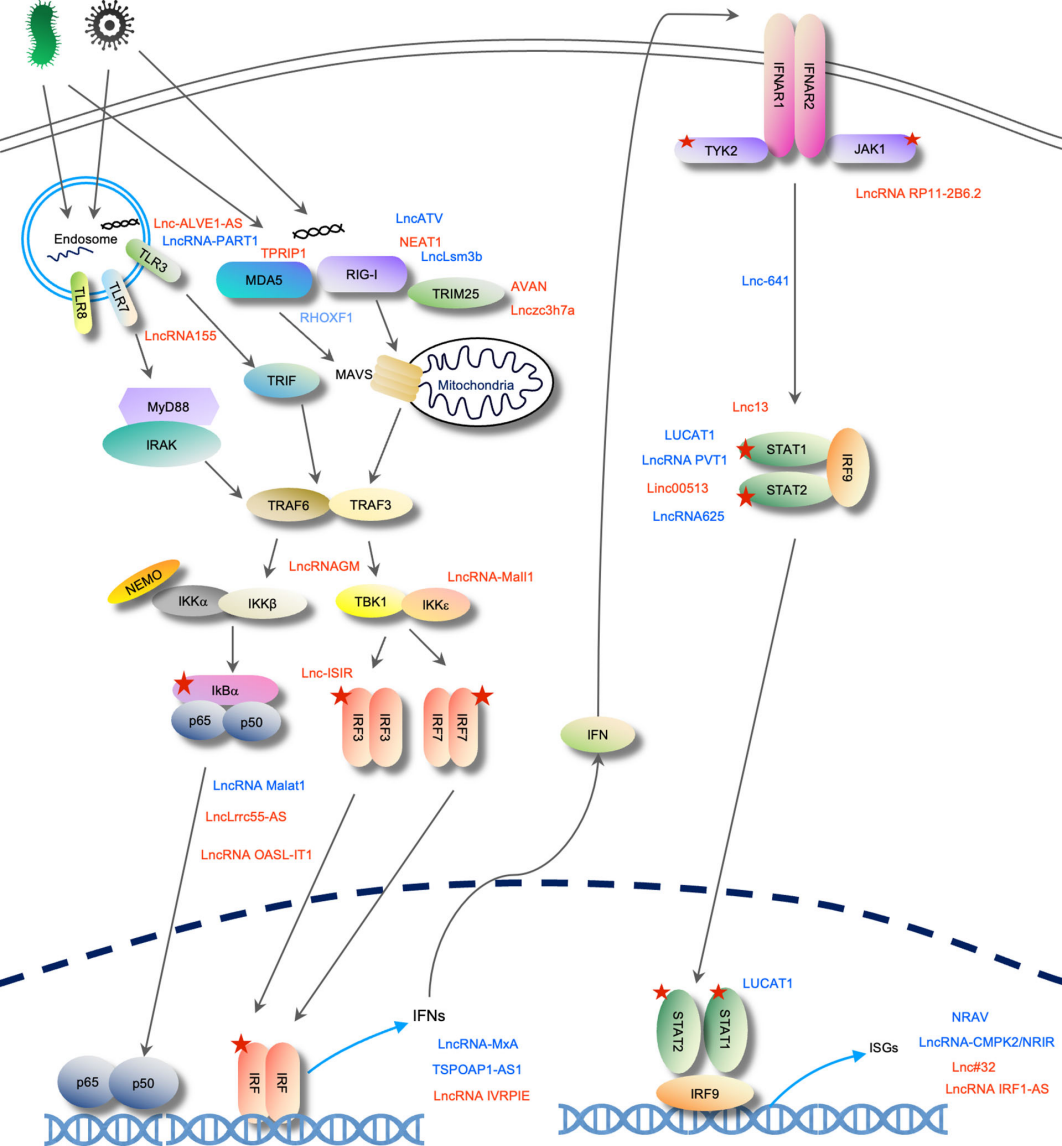
A summary of lncRNAs discussed in this review. From IFN signaling activation to ISG transcription, discussed lncRNAs are positioned at sites of action. Five-sided star represents phosphorylation modification. LncRNA in blue represents negative regulators; lncRNA in red is positive regulators for IFN signaling.

Here, we summarized the interferon signaling pathway mediated by nucleic acid-sensing receptors TLRs and RLRs, the biology of lncRNAs, and the regulatory roles of lncRNAs on IFN signaling. LncRNA acts on multiple steps, from PRRs’ sensing of ligand to the transcription of ISGs (**Figure 7**).

It is interesting to note that many publications cited in this review came out in recent years. Part of the reasons for this delay may be due to the difficulties to identify new lncRNAs. For now, many groups do so based on expression differentiation analysis, rather than using function-guided screening strategies, for example, the screening of a virus’s host receptor. Questions related to newly identified lncRNAs always focus on the following: how it is identified, where is it localized, does it encode proteins, and what its function is. Mechanism study is harder for lncRNA because of the following reasons: 1) unlike protein-encoded genes that can be easily detected the expression by western blotting, ectopic expression of lncRNA is not easily validated for its expression, especially the conformation and structure. 2) unlike proteins having domains, which are relatively independent of each other in structure, lncRNAs are hard to do truncations for detailed study. In another word, full-length lncRNA may have a different structure in solutions compared to truncated ones, thus largely affecting the functions. 3) Structure of lncRNAs is hard to study. RNA molecules are flexible. The lack of sequence homology or unawareness of it makes it hard to category “domains” of lncRNAs, resulting in a situation that each lncRNA seems to be unique and its mechanism requires a *de novo* investigation.

Despite these, X-ray crystallography, NMR spectroscopy, sequence-based analysis, and others solve structures of some of these single-stranded RNA transcripts successfully (107). The structural analysis benefits our understanding of mechanisms of lncRNA activities, such as details of binding to substrates (108). Efforts in dissecting the nature of lncRNA thus, help to explain their important roles in various aspects of cellular metabolisms, proliferation, programmed cell death, and others (109–111).

The IFN signaling is critical for host defense against pathogens (112) and is shown to be required for optimal chemotherapies (113, 114). Its aberrant activation due to gene mutations leads to type-I interferonopathies (115). In all cases, manipulating the IFN signaling can be good. Thus, exogenously expressed lncRNAs have potentials to be applied as approaches to regulate IFN signaling in different scenarios. With the progress on *in vitro* transcription and RNA-delivery system study, the application of lncRNAs will become feasible.

## Author Contributions

XJ, WM, ZL, and XM wrote and revised the manuscript. XJ draw the figures. All authors contributed to the article and approved the submitted version.

## Funding

This work was supported by the Strategic Collaborative Research Program of the Ferring Institute of Reproductive Medicine (Grant FIRMC200501 to ZL) and the State Key Laboratory of Veterinary Etiological Biology, CAAS and the Guangdong Provincial Key Laboratory of Precision Medicine and Clinical Translation Research of Hakka Population (Grants SKLVEB2020KFKT001 and 2018B030322003KF03 to XM).

## Conflict of Interest

The authors declare that the research was conducted in the absence of any commercial or financial relationships that could be construed as a potential conflict of interest.

## Publisher’s Note

All claims expressed in this article are solely those of the authors and do not necessarily represent those of their affiliated organizations, or those of the publisher, the editors and the reviewers. Any product that may be evaluated in this article, or claim that may be made by its manufacturer, is not guaranteed or endorsed by the publisher.

## Glossary

**Table:**

IFN	interferon
RIG-I	retinoic acid-inducible gene I
MDA5	melanoma differentiation-associated protein 5
TLR	toll-like receptor
dsRNA	double-stranded RNA
ADAR1	adenosine deaminase
RNA-specific 1	LOF
loss-of-function	ncRNA
non-coding RNA	lncRNA
long non-coding RNA	PRR
patten-recognition receptor	PAMP
pathogen-associated molecular pattern	DAMP
damage-associated molecular pattern	LPS
lipid polysaccharide	ssRNA
single-stranded RNA	cGAS
cyclic GMP-AMP synthase	cGAMP
cyclic GMP-AMP	STING
stimulator of interferon genes	MAVS
mitochondrial antiviral-signaling protein	TRIF
TIR-domain-containing adapter-inducing interferon-βγMyD88	myeloid differentiation primary response 88
IRF3	interferon regulatory factor 3
TBK1	TANK binding kinase 1
NEMO	NF-κB essential modulator
IKK	IκB kinases
IFNAR	IFN-α/β receptor
JAK	Janus kinase
STAT	signal transduction factors and activators of transcription
ISGF3	IFN-stimulated gene factor 3
ISG	IFN-stimulated gene
TRIM5α	tripartite motif-containing 5 alpha isoenzyme
OAS	oligoadenylate synthetase
circRNA	circular RNA
siRNA	small interfering RNA
miRNA	microRNA
kb	kilobases
MRE	miRNA response factor
snoRNA	small nucleolar RNA
BACE1	beta-site amyloid precursor protein cleaving enzyme 1
AD	Alzheimer’s disease
EMT	epithelial-mesenchymal transition
RLR	RIG-I-like receptor
SFPQ	splicing factor proline- and glutamine-rich protein
HTNV	hantaan virus
HUVEC	human umbilical vein endothelial cell
RNF135	ring finger protein 135
TRIM25	tripartite motif-containing protein 25
VSV	vesicular stomatitis virus
nt	nucleotide
SeV	Sendai virus
EMCV	encephalomyocarditis virus
HSV-1	herpes simplex virus-1
IAV	influenza A virus
ADV	adenovirus
HCV	hepatitis C virus
RIP	RNA immunoprecipitation
5-Aza-CdR	5-Aza-2′-deoxycytidine
CEF	chicken embryo fibroblast
ERV	endogenous retrovirus
PTP1B	protein tyrosine phosphatase-1B
PC	prostate cancer
GSTM1	Glutathione S-transferase mu 1
OPTN	optineurin
siRNA	small interfering RNA
Lrrc55	leucine rich repeat containing 55
PME-1	phosphatase methylesterase 1
PP2A	phosphatase 2A
FA-CLIP	formaldehyde-crosslinked RNA immunoprecipitation
Fli-1	Flightless-1
ZIKV	Zika virus
MAPK	mitogen-activated protein kinase
TDP43	transactive response DNA binding protein 43
Rbck1	Ring-B-box-coiled-coil protein interacting with protein kinase C-1
ChIP	Chromatin immunoprecipitation
PBMC	peripheral blood mononuclear cell
LN	lupus nephritis
SOCS1	suppressor of cytokine signaling 1
TYK2	tyrosine kinase 2
SLE	systemic lupus erythematosus
hnRNP	heterogeneous nuclear ribonucleoprotein
HDAC1	histone deacetylase 1
NuRD	nucleosome remodeling and deacetylating
CVB5	Coxsackie Virus B5
PCBP2	poly[rC] binding protein 2
3′-UTR	3′-untranslated region
PRV	pseudorabies virus
ESCC	esophageal squamous cell carcinoma
FISH	fluorescence *in situ* hybridization
hMDDC	human monocyte-derived dendritic cell
IFI16	interferon gamma inducible protein 16
IVRPIE	inhibiting IAV replication by promoting IFN and ISGs expression
RSV	respiratory syncytial virus
TSS	transcription start site
H3K4me3	tri-methylation of lysine 4 on histone H3
ILF3	interleukin enhancer binding factor 3
DHX9	DExH-Box helicase 9
LUARIS	lncRNA upregulator of antiviral response interferon signaling
CRE	cAMP response element
MDRV	Muscovy Duck reovirus
ZONAB	zonula occludens 1-associated nucleic acid binding protein
PCNA	proliferating cell nuclear antigen
NRIR	negative regulator of interferon response
